# IVL-SYNTHSFM-v2: A synthetic dataset with exact ground truth for the evaluation of 3D reconstruction pipelines

**DOI:** 10.1016/j.dib.2019.105041

**Published:** 2019-12-23

**Authors:** Davide Marelli, Simone Bianco, Gianluigi Ciocca

**Affiliations:** DISCo—Department of Informatics, Systems and Communication, University of Milano - Bicocca, viale Sarca 336, 20126, Milano, Italy

**Keywords:** Structure from Motion (SfM), 3D reconstruction, Blender, Realistically rendered images

## Abstract

This article presents a dataset with 4000 synthetic images portraying five 3D models from different viewpoints under varying lighting conditions. Depth of field and motion blur have also been used to generate realistic images. For each object, 8 scenes with different combinations of lighting, depth of field and motion blur are created and images are taken from 100 points of view. Data also includes information about camera intrinsic and extrinsic calibration parameters for each image as well as the ground truth geometry of the 3D models. The images were rendered using Blender. The aim of this dataset is to allow evaluation and comparison of different solutions for 3D reconstruction of objects starting from a set of images taken under different realistic acquisition setups.

**Table:** Specifications Table

Subject	Computer Vision and Pattern Recognition
Specific subject area	3D reconstruction from images
Type of data	Image CSV file 3D model
How data were acquired	Images of some virtual scene portraying the 3D models were rendered using Blender. The camera pose parameters were exported in plain text files.
Data format	Raw
Parameters for data collection	3D scenes with different subjects, varying lighting conditions, depth of field and motion blur; acquired by a moving camera from various poses.
Description of data collection	4000 synthetic images of five different 3D scenes, were rendered by means of Blender. Each scene was rendered using different camera poses, lighting condition, depth of field and motion blur. Cameras calibration parameters, intrinsic and extrinsic, were collected for each rendered image.
Data source location	Institution: University of Milano – Bicocca City: Milano Country: Italy
Data accessibility	Data are available on Mendeley Data at https://doi.org/10.17632/fnxy8z8894.1
Related research article	S. Bianco, G. Ciocca, D. Marelli. Evaluating the Performance of Structure from Motion Pipelines. J. Imaging 2018, 4, 98. https://doi.org/10.3390/jimaging4080098.

**Table:** 

**Value of the Data** • The data can be used to evaluate and compare 3D reconstructions of single objects from multiple images obtained using various techniques. The different lighting and acquisition conditions are introduced to make the dataset suitable to test the robustness of the reconstruction pipelines on different image acquisition setups. • The data is of interest to researchers that would like to test and compare various 3D reconstruction methods to check the results of different approaches to the reconstruction of single objects. Can be used to assess the performance of state-of-the-art methods as well as to evaluate and compare new techniques. • The data allow to evaluate the impact of variations in illumination conditions, depth of field and motion blur on the reconstruction pipelines. • The data can be used to determine how a 3D reconstruction method reacts when used on images of objects with differences in size, geometry and texture details. • The data contains information about camera intrinsic and extrinsic calibration parameters that allow precise camera positioning, reconstruction estimation, and evaluation. This is highly relevant in the case of evaluation of reconstructions made by techniques that assume unknown camera poses (e.g. Structure from Motion) and reconstructions pipelines that require known camera poses, such as Multi View Stereo (MVS). • The synthetic data generation process allows to provide along with the images precise information about camera positioning and geometry ground truth, such level of ground truth's accuracy allows precise evaluation of the reconstructed 3D geometry.

## 1 Data

**Fig. 1:**
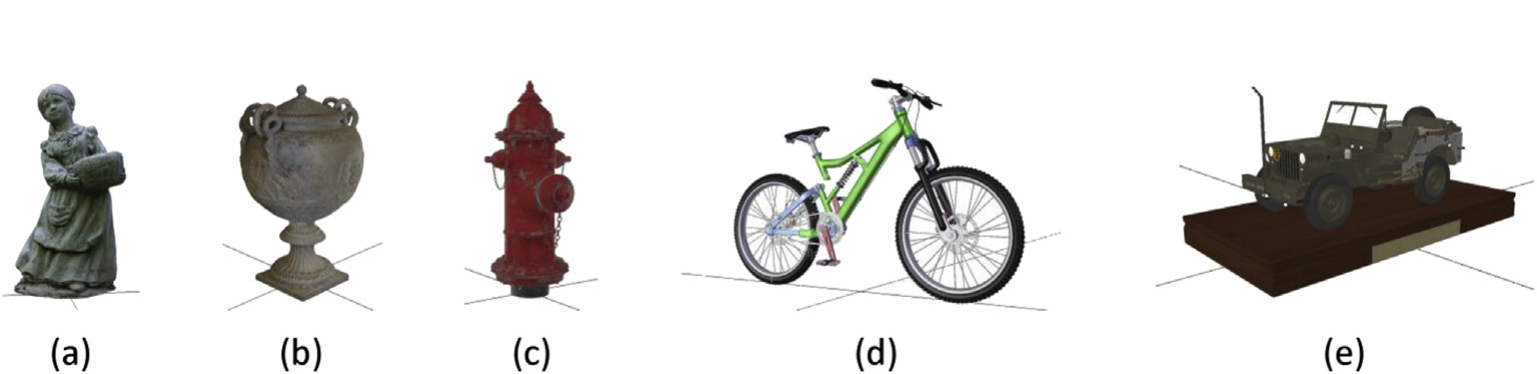
3D models used for synthetic data generation: (a) Statue [4]. (b) Empire Vase [5]. (c) Hydrant [6]. (d) Bicycle [7]. (e) Jeep [8].

The dataset is publicly available for download at [1]. The images in this dataset are generated from the five 3D models shown in Fig. 1. Each model is placed in a reference scene and is rendered under different lighting and camera conditions. For each object, 8 scenes are created and images are taken from different points of view. Each scene is composed of a set of 100 captured images. The list of the image sets, along with the acquisition setup, is shown in Table 1. The total number of images in the IVL-SYNTHSFM-v2 dataset is 4000.

**Table 1:** List of available images sets for each object in the dataset.

Set name / data subfolders	Lighting setup	Depth of field	Motion blur
fs	Sun, fixed position	No	No
fs-dof	Sun, fixed position	Yes, on all images	No
fs-mb	Sun, fixed position	No	Yes, on random images
fs-dof-mb	Sun, fixed position	Yes, on all images	Yes, on random images
ms	Sun, random position	No	No
ms-dof	Sun, random position	Yes, on all images	No
ms-mb	Sun, random position	No	Yes, on random images
ms-dof-mb	Sun, random position	Yes, on all images	Yes, on random images

The selected 3D models (Fig. 1) were chosen based on the different levels of geometry complexity and texture detail which translates into different levels of complexity for the reconstruction process. The Statue model is composed by 60k vertices, 295k for the Vase, 9k the Hydrant, 300k the Bicycle and 2335k for the Jeep model.

For each scene a Comma-separated values (CSV) file ‘scene.csv’ describes some parameters of the acquisition setup:

• scene_name: string, name of the acquisition setup, same as the 3D model name
• images_count: integer, number of images in each set, always 100
• unit_system: string, measurement unit system, always METRIC
• unit_length: string, length unit, always METERS
• scene_center_x,y,z: three floats, coordinate of the scene's center
• scene_ground_center_x,y,z: three floats, coordinate of the scene's ground center
• scene_width, depth,height: three floats, size of the scene along X, Y and Z axes
• mean_cam_dist_center: float, mean camera distance from the center of the scene
• mean_cam_dist_obj: float, mean camera distance from the object's surface, computed as the distance between the camera and the first point of intersection with the object along the camera's look-at direction
• mean_cam_height: float, mean camera height from scene's ground

All the images rendered for each 3D scene are available as JPG files of resolution 1920 × 1080 pixels. The images were acquired using a perspective virtual camera with a 35 mm focal length and 18 × 32 mm sensor, this information can also be found in the EXIF metadata (version 2.3) of each image.

For each set of images is also included a CSV file ‘cameras.csv’ containing for each image information about: camera position, camera rotation, camera look-at direction, depth of field, motion blur and sun lighting position. All the vectors and quaternions are defined in a right-handed coordinate reference system defined by X growing right, Z growing upwards, Y growing forward. The names of the fields are included in the first line of the file and are structured as follow:

• image_number: four digits image number, same as image filename without extension
• cam_position_x,y,z: three floats, camera position vector
• cam_rotation_w,x,y,z: four floats, camera rotation quaternion
• cam_lookat_x,y,z: three floats, camera look-at direction vector
• depth_of_field: boolean, ‘True’ if image rendered with depth of field enabled, ‘False’ otherwise
• motion_blur: boolean, ‘True’ if image rendered with motion blur enabled, ‘False’ otherwise
• sun_azimuth: float, azimuth angle in radians of the sun lamp illuminating the scene
• sun_inclination: float, inclination angle in radians of the sun lamp illuminating the scene

Samples of entries that can be found in ‘cameras.csv’ files are presented in Table 2.

**Table 2:** Samples of data in cameras.csv files.

Image number	Position X	Position Y	Position Z	Rotation W	Rotation X	Rotation Y	Rotation Z	Look-at X	Look-at Y	Look-at Z	Depth of field	Motion blur	Sun azimuth	Sun inclination
0001	7.007345	−6.647154	3.905988	0.765872	0.507658	0.218024	0.328920	−0.667915	0.634178	−0.389497	True	True	−2.212487	1.049574
0002	6.736142	−7.548378	4.048048	0.777758	0.517985	0.197374	0.296358	−0.614036	0.688747	−0.385470	True	False	−1.935121	0.965231
0003	6.036173	−7.806846	3.986951	0.788677	0.523167	0.178511	0.269107	−0.563151	0.729142	−0.388861	True	False	0.000000	1.744416
0004	5.710015	−8.167952	3.760057	0.788783	0.536442	0.168756	0.248139	−0.532448	0.762523	−0.367503	True	False	−0.531763	1.270089
0005	5.202869	−8.192430	4.075651	0.803880	0.525362	0.152563	0.233443	−0.490569	0.773427	−0.401438	True	True	−0.476696	1.311158

Along with the images and the CSV files the 3D models are made available as WaveFront Object (.obj) files describing the sole geometry of the objects. In addition, is included the original archive (.zip) containing the 3D model that also defines the materials and textures. This allow evaluation and comparison of different solution for 3D reconstruction of objects starting from a set of images as explained in Refs. [2,3].

## 2 Experimental design, materials, and methods

**Fig. 2:**
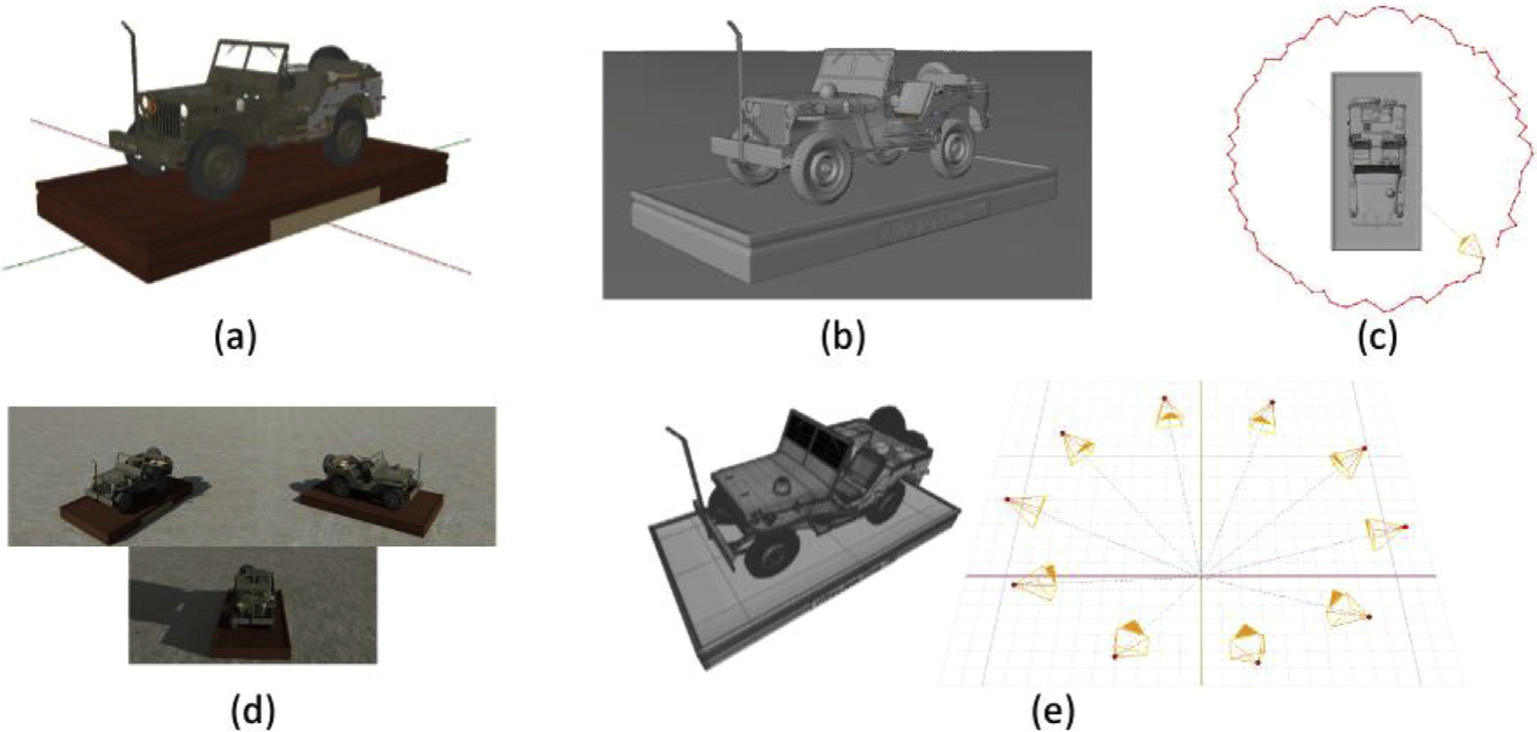
Example of data generation steps for the Jeep model: (a) 3D model. (b) Scene setup with main object, floor surface and lights. (c) Camera animation around the object. (d) Images rendering. (e) 3D model geometry and camera pose ground truth export.

The dataset was created using Blender as 3D modeling and rendering software. For each scene portraying a single object, a 3D model is placed in the center of the scene leaning on a plane, a sun lamp is then used to light up the environment. To have a more realistic scene the floor is textured with a concrete looking material and a sky with procedural clouds is created. The scene is then observed from different viewpoints by a moving perspective camera. The camera uses a sensor of size 18 × 32 mm and a 35 mm focal length, all the images are acquired at resolution 1920 × 1080 pixels. To obtain a complete coverage of the object the camera moves in a circle around the vertical axis at the scene's center, depending on the complexity and size of the object the movement can be a single circle or two circles at different height. To simulate realistic manual acquisition the camera position is randomized by 5% of the acquisition points sampled on the movement circle. A sample scene setup is visible in Fig. 2.

For each of these scenes portraying different objects 8 sets are acquired under different conditions of lighting, depth of field and motion blur. For the sets that make use of a moving sun, the sun lamp is placed at a random position for each image; this is intended to simulate the acquisition during different hours of the day. The sun position is randomized along a semicircular path and kept consistent within the sets about the same object. The depth of field is applied to all images of the images sets that make use of it. Finally, in the sets that make use of motion blur the effect is introduced randomly on approximately 33% of the images.The different setups for each object can be used to evaluate the performances of reconstruction pipelines under different light conditions and the robustness to depth of field and motion blur.

**Fig. 3:**
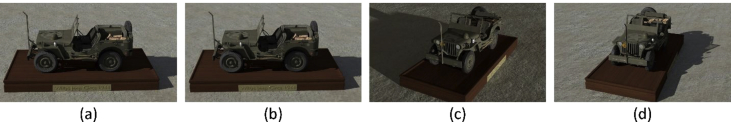
Sample of rendered images of the Jeep model. (a) image from the ‘fs’ set. (b) image from the ‘fs-dof-mb’ set. (c, d) images from the ‘ms’ set.

The images are rendered using Cycles, the Blender's path-tracing render engine, that simulates physics-based light interactions and allows generation of photo-realistic images. Samples of rendered images are visible in Fig. 3.

The geometry ground truth model (.obj files) can be used to evaluate the quality of the reconstruction by means of the distance between the reconstructed point cloud or mesh and the ground truth model. The information about camera poses can be used to evaluate the precision of the estimated poses in the case of methods that recover these parameters as part of the reconstruction process. Such evaluation can be done in terms of distance between camera position and difference in orientation. Further details on how to use the camera parameters can be found in Ref. [2]. Furthermore, camera pose parameters can be used to run 3D reconstructions with techniques that require known camera positions and rotations.
